# Diet-Induced Alterations in Total and Metabolically Active Microbes within the Rumen of Dairy Cows

**DOI:** 10.1371/journal.pone.0060978

**Published:** 2013-04-10

**Authors:** Abderzak Lettat, Chaouki Benchaar

**Affiliations:** Agriculture and Agri-Food Canada, Dairy and Swine Research and Development Centre, Sherbrooke, Quebec, Canada; U. S. Salinity Lab, United States of America

## Abstract

DNA-based techniques are widely used to study microbial populations; however, this approach is not specific to active microbes, because DNA may originate from inactive and/or dead cells. Using cDNA and DNA, respectively, we aimed to discriminate the active microbes from the total microbial community within the rumen of dairy cows fed diets with increasing proportions of corn silage (CS). Nine multiparous lactating Holstein cows fitted with ruminal cannulas were used in a replicated 3×3 Latin square (32-d period; 21-d adaptation) design to investigate diet-induced shifts in microbial populations by targeting the rDNA gene. Cows were fed a total mixed ration with the forage portion being either barley silage (0% CS), a 50∶50 mixture of barley silage and corn silage (50% CS), or corn silage (100% CS). No differences were found for total microbes analyzed by quantitative PCR, but changes were observed within the active ones. Feeding more CS to dairy cows was accompanied by an increase in *Prevotella* rRNA transcripts (*P* = 0.10) and a decrease in the protozoal rRNA transcripts (*P*<0.05). Although they were distributed differently among diets, 78% of the amplicons detected in DNA- and cDNA-based fingerprints were common to total and active bacterial communities. These may represent a bacterial core of abundant and active cells that drive the fermentation processes. In contrast, 10% of amplicons were specific to total bacteria and may represent inactive or dead cells, whereas 12% were only found within the active bacterial community and may constitute slow-growing bacteria with high metabolic activity. It appears that cDNA-based analysis is more discriminative to identify diet-induced shifts within the microbial community. This approach allows the detection of diet-induced changes in the microbial populations as well as particular bacterial amplicons that remained undetected using DNA-based methods.

## Introduction

It is well established that culture-based methods greatly underestimate microbial population size and diversity. The ribosomal DNA (rDNA) gene is considered as a molecular clock and has been used as a means to decipher phylogenetic relationships and identify microbial species [1]–[2]. Consequently, rDNA-based methods have been being widely used and have greatly improved our knowledge of rumen microbial ecology [3]–[6]. However, rDNA-based methods are not specific to the metabolically active microorganisms because DNA may also originate from inactive and/or dead cells that are not involved in fermentation processes [7]–[8]. In contrast, several studies reported a linear correlation between ribosomal RNA (rRNA) concentrations and microbial activity [9]–[12]. Moreover, numerous reports showed that rRNA-based methods are more discriminative for monitoring microbial shifts as compared to rDNA-based methods [7], [13]–[14], enabling its utilization as a marker for metabolically active microbes.

Dietary manipulation is used to improve animal performance and decrease the impact of ruminant production on the environment (e.g., reduced methane emissions and nitrogen excretion). Thus, the formulation of the diet can have a significant effect on ruminal microbiota [15]. However, existing microbial records do not reflect the entire microbial population and further research is needed to assess how active microbes are affected by dietary changes.

In the rumen, starch fermentation favours propionate production at the expense of acetate and reduces ruminal pH. Consequently, the availability of hydrogen is lowered which can inhibit the growth and the activity of rumen methanogens [16]–[17]. Rumen protozoa are also often decreased in ruminants fed high-starch diets, which also reduces the transfer of hydrogen from protozoa to methanogens [18]–[19]. Because corn silage contains more starch than barley silage, increasing its proportion in the diet can modulate rumen microbial population by enhancing growth and activity of *Prevotella* that promotes propionate production [20]–[21], and decreasing the protozoal population. These changes would make the rumen environment less favorable to methanogenic *Archaea* and would result in lower methane energy losses in cows fed corn silage-based diets as compared to cows fed barley silage-based diets.

The aim of this study was to use RNA and DNA to discriminate the metabolically active microbes (i.e, responsible for feed degradation) from the total microbial community (i.e., active and inactive cells) in the rumen of dairy cows fed increasing proportions of corn silage. For this purpose, the quantitative PCR (qPCR) and amplicon length heterogeneity PCR (LH-PCR) techniques were used. LH-PCR is a valuable fingerprinting method based on the natural length variation in the *rrs* gene and has been successfully applied to monitor bacterial and archeal dynamics in different ecosystems [7], [14], [22]–[23]. This technique is much easier, cheaper and less time-consuming than DGGE (denaturing gradient gel electrophoresis) and TRFLP (terminal restriction fragment length polymorphism), because it does not require polyacrylamide gel handling and enzyme digestion [24].

## Materials and Methods

### Ethics Statement

All animal procedures were conducted with the approval (Permit Number 368) of the Animal Care Committee of the Dairy and Swine Research and Development Center (Agriculture and Agri-Food Canada, Sherbrooke, Quebec, Canada) and were in accordance with the guidelines of the Canadian Council on Animal Care (1993).

### Dairy Cows and Experimental Diets

Nine multiparous (3.3±0.6 parity; mean ± SD) lactating Holstein cows fitted with ruminal cannulas (10 cm, Bar Diamond Inc., Parma, ID, USA) were used in a replicated 3×3 Latin square (32-d period; 21-d adaptation). The ruminal surgery was performed on all cows 4 months before starting the experiment according to the technique described in Duffield [25]. The cows averaged 114±33 days in milk (mean ± SD) at the start of the experiment with an average body weight of 707±49 kg (mean ± SD), and 47±2.6 kg/d of milk yield (mean ± SD). No antibiotics or antimicrobials were fed to cows 6 months prior the experiment. Cows were fed twice daily (9.00 a.m. and 7.00 p.m.) for *ad libitum* intake a total mixed ration (60∶40 forage:concentrate ratio, DM basis) with the forage portion being either barley silage (**0% CS**), a 50∶50 mixture of barley silage and corn silage (**50% CS**), or corn silage (**100% CS**). The cows were kept in individual stalls and had free access to water during the experiment. The diet (Table S1) contained (on dry matter basis) 167, 161, and 159 g/kg crude protein; 244, 221, and 186 g/kg acid detergent fiber; and 166, 206, and 256 g/kg starch for 0% CS, 50% CS, and 100% CS treatment, respectively.

### Rumen Sample Collection

On d-21 of each experimental period, 2 kg of total ruminal content were collected before the a.m. feeding from the anterior dorsal, anterior ventral, medium ventral, posterior dorsal, and posterior ventral locations within the rumen of each cow. After homogenization of the ruminal content collected, ∼200 g sub-samples were homogenized on ice using a PT 10/35 Polytron homogenizer (Kinematica GmbH, Bohemia, NY, USA) at speed 6, for two 2-min cycles with 1 min rest in ice between cycles. Subsequently, representative aliquots of 3 g were stored at −80°C pending nucleic acid extraction. Meanwhile, sub-samples of total ruminal content (0.3 to 0.5 g) were dried at 100°C for 72 h for the determination of dry matter (DM) concentration.

### Total Nucleic Acid Extraction and cDNA Synthesis

Total nucleic acid (RNA and DNA) was co-extracted from the frozen samples using the FastRNA® Spin Kit (MP Biomedicals, Solon, OH, USA) according to the manufacturer instructions with modification. Briefly, ∼250 mg of frozen ruminal content were weighed in tubes containing silica beads. For cell lysis, samples were subjected twice to a 1-min pulse with 1 min cooling in ice between the two pulses, using the Mini-Beadbeater-8^TM^ (BioSpec Products, Bartlesville, OK, USA). The nucleic acid purity was verified by electrophoresis on agarose gel (1.2% wt/vol) and staining with ethidium bromide. The total nucleic acid aliquot (100 µL) was divided into 2 equal aliquots. To obtain RNA, DNA was digested in one of the two aliquots using the RNase-free DNase Set (Qiagen Inc., Toronto, ON, Canada) according to the manufacturer recommendations. The yield and the purity of DNA and RNA aliquots were assessed by optical density measurement (NanoDrop ND-1000 spectrophotometer, Thermo Fisher Scientific, Wilmington, DE, USA). Absorbance intensity at 260 nm was used to assess the concentration of nucleic acid in 1 µL of sample while sample purity was checked at 260/280 and 260/230 ratios. To ensure that all DNA was digested, qPCR assays were run on RNA samples and no signal was observed which proves absence of DNA contamination.

For cDNA synthesis, the extracted RNA was reverse transcribed using the AMV reverse transcriptase (Promega, Madison, WI, USA) according to the manufacturer instructions with minor modifications. Briefly, 1 µg of RNA was mixed with 0.5 µg of random primers and sterilized water to achieve a final volume of 15 µL. The mixture was brought to 70°C for 5 min followed by 10 min on ice to allow annealing. Then, a 25 µL buffer mixture containing 1× AMV buffer, 1 µM of each dNTP, 1.6 U of RNasin® and 3.6 U of AMV reverse transcriptase (Promega, Madison, WI, USA) was added. Reverse transcription was carried out at 37°C for 1 h using the C1000 thermal cycler (Bio-Rad).

### Protozoa, *Bacteria* and *Archaea* Quantification by qPCR

In this study, we quantified protozoa, methanogenic *Archaea*, total bacteria, and specific bacteria that were selected based on their role in ruminal fermentation processes (i.e., starch and fiber degradation), utilization and/or production of hydrogen. Protozoa are important hydrogen-producers within the rumen while the methanogenic *Archaea* utilize the hydrogen for methane production [16], [26]. The examined bacteria included *Prevotella* genus, which is the dominant amylolytic bacterial group and an efficient hydrogen utilizer for propionate production, and the non-hydrogen-producing cellulolytic bacterium *Fibrobacter succinogenes* to determine wether the fibrolytic activity is impaired by the diets used [20]–[21], [27].

The SYBR green chemistry-based qPCR was carried out using the StepOnePlus™ Real-Time PCR System (Life Technologies, Mississauga, ON, Canada). Protozoa, total and selected bacteria (*Prevotella* genus, *Fibrobacter succinogenes*) were quantified by targeting the small ribosomal subunit (*rrs*) gene, while the *rrs* and methyl coenzyme-M reductase (*mcrA*) genes were used for methanogenic *Archaea* detection [28]. The qPCR mixture was composed of 0.75X SYBR Premix Ex Taq II (Clontech Laboratories Inc., Madison, WI, USA), 40 ng of DNA or cDNA template, and 0.25 µM of each forward and reverse primer except for *Archaea* and *Prevotella* for which 0.3 and 0.5 µM were used, respectively. Each reaction was run in triplicate in 96-well plates (Life Technologies, Mississauga, ON, Canada). The primer sequences and amplification programs used are summarized in Tables S2 and S3. An absolute quantification of bacteria was performed using specific *rrs* DNA fragments from, *F. succinogenes* S85 (ATCC 19169) and *P. bryantii* B14 (DSM 11371). For protozoa, *rrs* DNA standards were prepared as described in Sylvester et al. [29]. For methanogenic *Archaea*, *rrs* and *mcrA* DNA fragments from *Methanobrevibacter smithii* (DSM 861) were used. Only qPCR assays that fell within the range from 90 to 110% of efficiency and showing an *r* ^2^≥0.99 were considered for further analysis.

### LH-PCR Fingerprints of *Bacteria* (*rrs* gene)

According to the method developed by Suzuki et al. [23], length heterogeneity PCR, based on natural length variation in the *rrs* gene (LH-*rrs*) was used to fingerprint the bacterial community. Briefly, in a final volume of 25 µL, the LH-*rrs* mixture was composed of 40 ng of DNA and cDNA template, 0.5 µM of each forward and reverse primer (Life Technologies, Mississauga, ON, Canada), 0.1 mM of dNTPs (Promega, Madison, WI, USA), 1× Taq buffer, 1.5 mM MgCl_2_ and 0.625 U of Taq polymerase (BioShop Inc., Burlington, ON, Canada) using a C1000 thermal cycler (Bio-Rad). The primer sequences and amplification programs used are summarized in Tables S2 and S3. After PCR amplification, capillary electrophoresis was performed on the DNA and cDNA samples as previously described [22], [30]. Briefly, 1 µL of template was mixed with 12.34 µL of Hi-Di Formamide (Life Technologies, Mississauga, ON, Canada) and 0.06 µL of GeneScan™ 500 LIZ® Size Standard (Life Technologies, Mississauga, ON, Canada) and the resulting mixture was heated at 95°C for 5 min then chilled on ice. Capillary electrophoresis was performed for 40 min in the GeneScan mode on an ABI Prism 310 47-cm capillary DNA sequencer using POP-4 polymer (Life Technologies, Mississauga, ON, Canada). The GeneMapper® software (Life Technologies, Mississauga, ON, Canada) was used for fingerprints analysis.

### Statistical Analyses

Data were analysed using the PROC MIXED of SAS (SAS Institute Inc., Cary, NC). Data were assessed for normality and logarithmically (log_10_) transformed prior to statistical analysis. The statistical model included treatment and period as fixed effects and square and cows within square as random effects. Differences between treatments were declared significant at *P*≤0.05 using the Tukey correction for multiple comparisons, and tendencies were discussed when 0.05<*P*≤0.10. The principal component analysis (PCA) was performed on the LH-*rrs* fingerprints using the PRINCOMP procedure of SAS with the COV option. Only the first two principal components were plotted. Indicator Species Analysis (ISA) and Multi-Response Permutation Procedure (MRPP) were performed using the PC-ORD software [31].

## Results and Discussion

### Microbial Populations

In this study, DNA was used as an indicator of microbial density whereas cDNA was used to estimate microbial growth and activity. Regarding the total microbial community (i.e., DNA samples), bacterial, archaeal and protozoal rRNA copies remained similar (*P*>0.10) among diets (Table 1). In contrast, quantification of the metabolically active microbes (i.e., cDNA samples) revealed a diet effect on the microbial populations. Indeed, feeding the 50% CS and 100% CS diets tended (*P* = 0.10) to increase the number *Prevotella* spp. rRNA transcripts compared to cows fed 0% CS diet. Meanwhile, the protozoal rRNA transcripts were reduced (*P*<0.05) in cows fed the 100% CS diet compared to those fed the 0% CS diet. These effects are likely due to the greater starch supply provided by increased proportions of CS in the diet (166, 206 and 256 g/kg for 0% CS, 50% CS and 100% CS diets, respectively). A greater starch supply accompanied by a decrease in ruminal pH and a shift of volatile fatty acids production towards more propionate has been often associated with an increase in *Prevotella* density and a decrease in protozoa numbers [4], [32]–[33]. No diet effect (*P*>0.10) was observed on methanogenic activity and growth of total bacteria, *F. succinogenes* and methanogenic *Archaea*.

**Table 1:** Abundance of *rrs* and *mcrA* gene copies and transcripts in total ruminal content of lactating cows (*n* = 9) fed diets with 0, 50 or 100% corn silage (CS)^1^.

	Treatments				
	0% CS	50% CS	100% CS	SEM	Treatment *P-*value
	Number of *rrs* and *mcrA* gene copies				
Total microbes (DNA)					
*Bacteria*	11.80	11.95	11.87	0.09	0.25
*Prevotella spp*.	10.67	10.84	10.87	0.09	0.14
*F. succinogenes*	9.25	9.24	9.16	0.09	0.76
*Archaea* (*rrs*)	8.79	8.88	8.91	0.10	0.74
*Archaea* (*mcrA*)	8.42	8.39	8.50	0.13	0.24
Protozoa	8.89	8.82	8.89	0.13	0.20
	Number of *rrs* and *mcrA* transcripts				
Active microbes (cDNA)					
*Bacteria*	10.61	10.73	10.75	0.08	0.86
*Prevotella spp.*	9.71^A^	9.88^B^	9.89^B^	0.07	0.14
*F. succinogenes*	8.46	8.46	8.33	0.07	0.34
*Archaea* (*rrs*)	8.32	8.40	8.32	0.08	0.39
*Archaea* (*mcrA*)	5.52	5.51	5.53	0.10	0.86
Protozoa	8.63^a^	8.49^ab^	8.31^b^	0.10	0.01

1 Results are expressed as log_10_ gene copies/g DM of total ruminal content.

a, b, A, B Within a row, means without a common superscript differ significantly (*P*≤0.05) for lowercase letters or tended (0.05<*P*≤0.10) to differ for uppercase letters.

### Bacterial Diversity

The bacterial community structure was investigated using the LH-PCR fingerprint method that targeted the *rrs* gene. Accordingly, 54 and 56 raw picks were identified in cDNA and DNA samples, respectively. After an iterative process of standardization that eliminated peaks with low original percentage [34], 51 and 52 true comparable peaks, with a length ranging from 316 to 396 base pairs (bp), were identified in cDNA and DNA samples, respectively.

**Figure 1:**
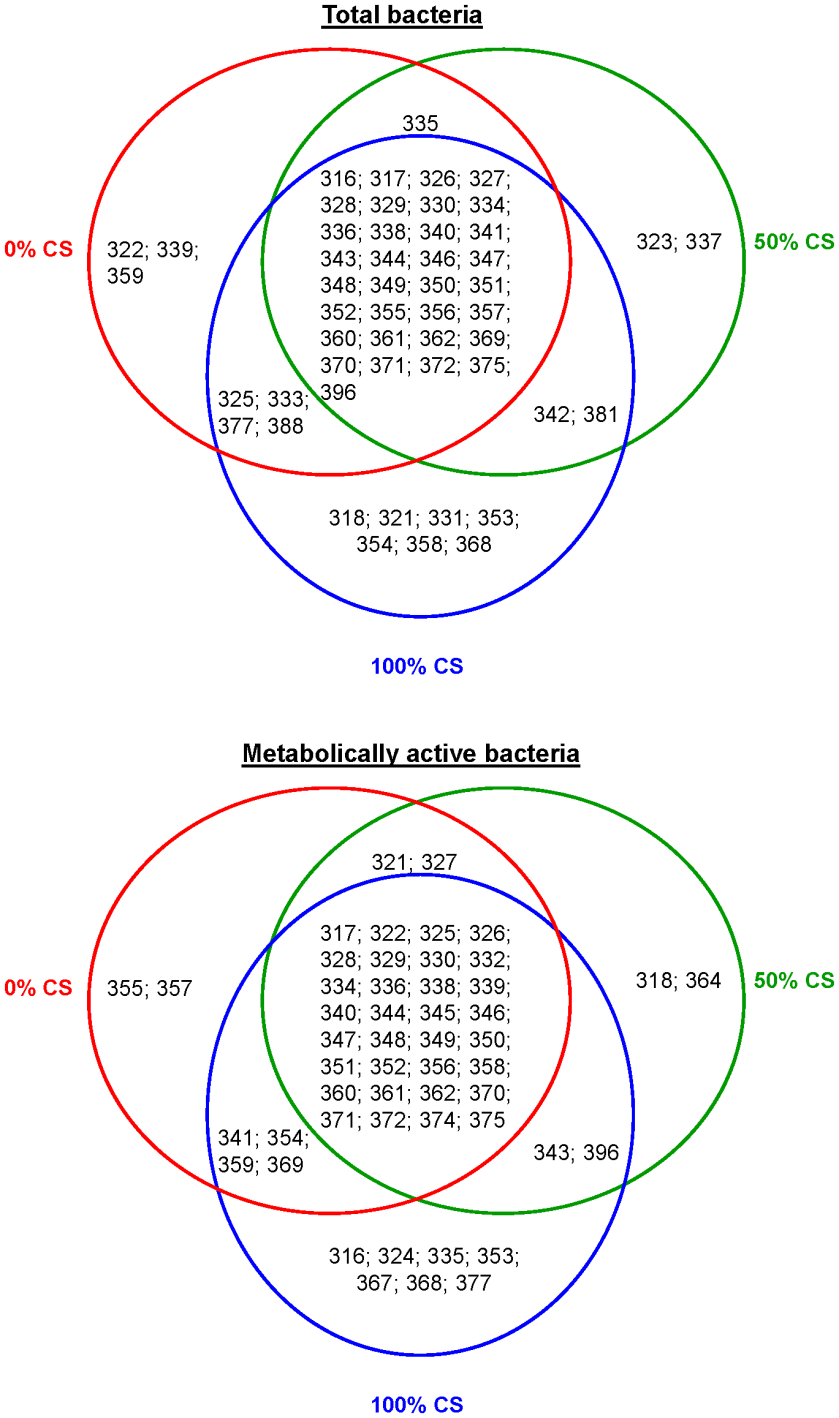
Venn Diagrams of bacterial LH-*rrs* amplicons representing shared and diet-specific amplicons that compose the total and the metabolically active bacterial communities. Total ruminal content (*n* = 9 cows for each diet) was collected before the a.m. feeding from dairy cows fed 0, 50 or 100% corn silage (CS).

Within the total bacterial community, the most abundant amplicons were the 336-, 328-, 344-, 375-, and 357-bp ones (found in 100, 96, 96, 93 and 93% of DNA samples; data not shown). According to the Venn diagram representation (Figure 1), 33 amplicons were common to the three diets, 1 amplicon (335-bp) shared between the 0% CS and 50% CS diets, 2 (342- and 381-bp) between the 50% CS and 100% CS diets, and 4 (325-, 333-, 377-, and 388-bp) between the 0% CS and 100% CS diets. For diet-specific amplicons, 3 (322-, 339- and 359-bp) were exclusively found in cows fed 0% CS, 2 (323- and 337-bp) in cows consuming 50% CS, and 7 (318-, 321-, 331-, 353-, 354-, 358-, and 368-bp) in cows fed 100% CS diet. In contrast, important changes were observed in the structure of the metabolically active bacterial community as compared to the total one. Indeed, the 334-, 336- and 370-bp amplicons were the most present among the LH-*rrs* fingerprints (all found in 96% of cDNA samples; data not shown), irrespective of the diet fed. As shown by the Venn diagram representation (Figure 1), the 355- and 357-bp amplicons were specific to the 0% CS diet, the 318- and 364-bp to the 50% CS, whereas the 316-, 324-, 335-, 353-, 367-, 368-, and 377-bp ones were only found in cows fed the 100% CS diet. Thirty two amplicons were common to the three diets, 2 (321- and 327-bp) were shared between the 0% CS and 50% CS diets, 2 (343- and 396-bp) between the 50% CS and 100% CS diets, and 4 (341-, 354-, 359-, and 369-bp) between the 0% CS and 100% CS diets. Among all the amplicons found herein, 78% were shared between total bacteria and the metabolically active ones. Thus, they may constitute an active bacterial core that ensures digestion processes within the rumen. In contrast, the 323-, 331-, 337-, 342-, and 381-bp amplicons were specific to total bacterial community and were not found among the active community and may represent inactive cells including dormant or dead cells [35]. In contrast, the 324-, 332-, 345-, 364-, 367-, and 374-bp amplicons were specific to metabolically active bacteria, which means that their cell density was low within the bacterial community and were therefore, not detected using DNA-based techniques [8], [36].

**Figure 2:**
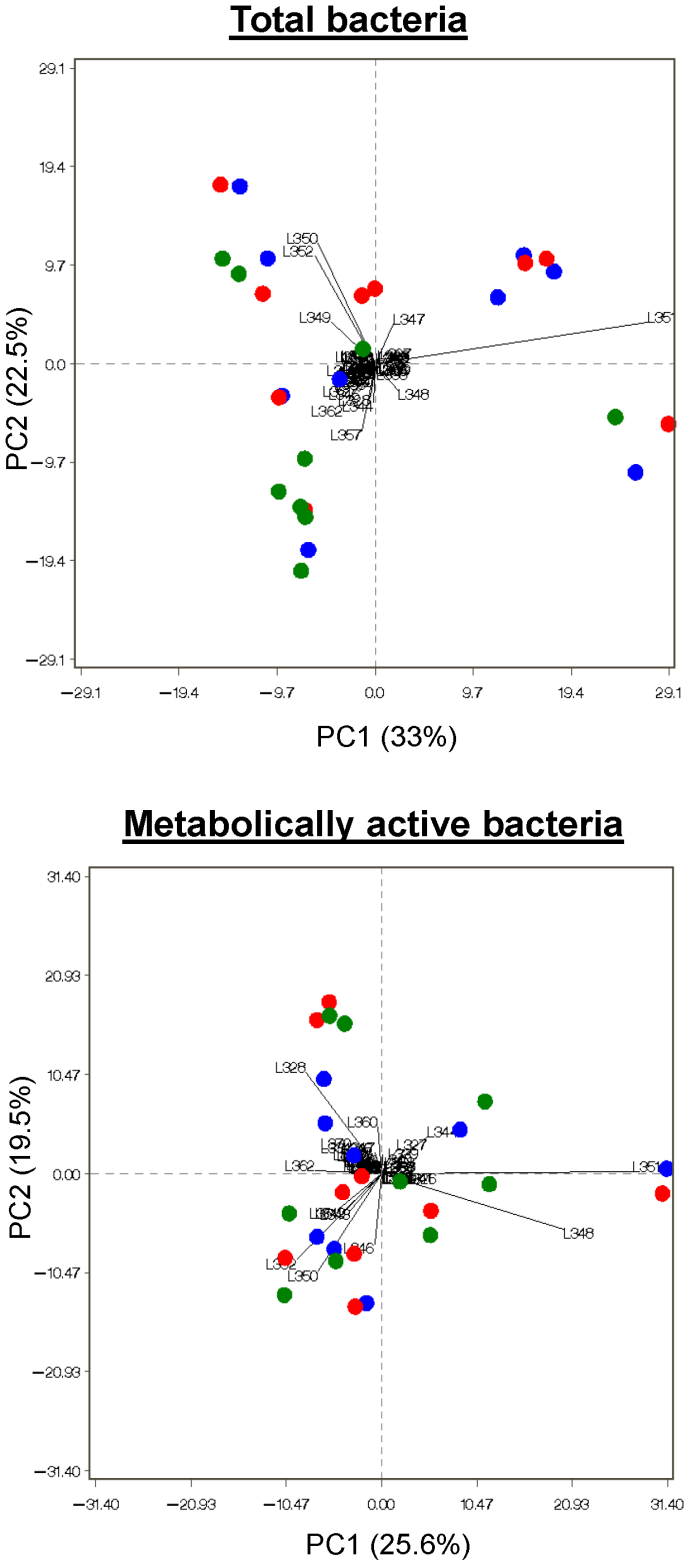
Principal component analysis ordination of bacterial LH-*rrs* amplicons that compose the total and the metabolically active bacterial communities. Total ruminal content (*n* = 9 cows for each diet) was collected before the a.m. feeding from dairy cows fed 0, 50 or 100% corn silage (CS). The percentage of total variance accounted for by each of the two principal components (PC1 and PC2) is shown in parentheses.

In an attempt to identify diet-induced differences in the bacterial structure due to feeding the cows increasing proportions of CS, the LH-*rrs* fingerprints retrieved from total and metabolically active bacteria were subject to a principal component analysis (PCA). For total and metabolically active bacteria, the two first principal components explained 55.5% and 45.1% of the total variance, respectively (Figure 2). The PCA showed that the 350-, 351- and 352-bp amplicons seem to be the most important within total bacterial community, while the 348- and 350-bp ones were the most significant within the metabolically active one. For both bacterial communities (total and active bacteria), none of the identified amplicons could be clearly related to a specific diet, which is in line with the qPCR results that did not reveal important changes in the bacterial community except *Prevotella* density that tended to increase with the 50% CS and 100% CS diets (Table 1). This was also supported by the MRPP analysis which was used to test for significance in difference among diets (*P*>0.10; data not shown), indicating that both total and metabolically active bacterial communities were unaffected by dietary changes. Similarly, the diversity indices that remained similar among diets for total and metabolically active bacteria (Table 2) indicate that the active bacterial community is as rich and diverse as the total bacterial one. This agrees with Reid et al. [35] who investigated bacterial diversity in the gut of the Huhu beetle larvae, but in contradiction with Portillo et al. [37] findings on bacterial colonization of Palaeolithic paintings in Spain. However, comparison of our results to those reported by Reid et al. [35] and Portillo et al. [37] should be taken with caution because of differences between the ecosystems (rumen *versus* larvae gut and paintings). To our knowledge, this is the first report on LH-PCR application to fingerprint the bacterial populations within the rumen.

**Table 2:** Bacterial diversity indices in lactating cows (*n* = 9) fed diets with 0, 50 or 100% corn silage (CS).

	Treatments				
	0% CS	50% CS	100% CS	SEM	Treatment *P*-value
Total bacteria (DNA)					
Richness	21.22	20.11	22.11	1.03	0.55
Diversity	2.46	2.43	2.53	0.08	0.55
Evenness	0.81	0.81	0.82	0.02	0.54
Active bacteria (cDNA)					
Richness	20.67	19.89	21.11	0.74	0.68
Diversity	2.59	2.58	2.61	0.06	0.88
Evenness	0.86	0.86	0.86	0.01	0.96

To further describe the diet-induced shifts in the total and the metabolically active bacterial communities, indicator species analysis (ISA) was performed on the LH-*rrs* fingerprints to determine whether a particular amplicon was significantly related to a specific diet [38]. Regarding the total bacteria, 5 indicator species were identified: the 347-bp in cows fed the 50% CS diet, and the 333-, 362-, 377-, and 396- bp in cows fed 100% CS diet (Table 3). For the metabolically active bacteria, the 355-bp amplicon was only found in cows fed 0% CS diet, whereas the 353-, 358- and 377-bp amplicons were dominant in cows consuming the 100% CS with the 353- and 377-bp being specific to this diet. Interestingly, ISA was more discriminative when applied to metabolically active bacteria since most of the amplicons identified were diet-specific (excluding the 358-bp that was shared by all diets), while those identified in the total bacterial community were common to at least 2 diets (Figure 1 and Table 3). Excluding the 377-bp amplicon that was found to be an indicator species for both total and metabolically active bacteria, ISA shows that the most active bacteria were not detected in DNA samples, which may indicate that these active bacteria are slow-growing microorganisms with high metabolic activity [12], [35].

**Table 3:** Indicator value (IV, %) of the bacterial LH-*rrs* fingerprints from lactating cows (*n = 9*) fed diets with 0, 50 or 100% corn silage (CS).

	Treatments				
Amplicons (bp)	0% CS	50% CS	100% CS	SD^2^	*P*-value^3^
Total bacteria (DNA)					
333	3	0	32	7.95	0.09
347	25	45	13	5.24	0.06
362	6	35	51	5.68	0.02
377	5	0	34	8.21	0.04
396	1	1	45	8.07	0.01
Active bacteria (cDNA)					
353	0	0	33	7.93	0.09
355	33	0	0	7.83	0.09
358	1	10	58	10.45	0.02
377	0	0	56	8.09	0.01

1 IV  =  relative abundance × relative frequency. Maximum IV for each amplicon is underlined.

2 Standard deviation.

3 IV were tested for significance using a Monte Carlo technique.

## Conclusions

This study investigated diet-induced shifts in total and metabolically active microbes within the rumen and revealed important differences. While no change was noticed within the total microbial community, monitoring the active microbes revealed a tendency towards an increase in *Prevotella* spp. rRNA transcripts and a decrease in those of protozoa when more CS was fed to dairy cows. Although some amplicons were found to be specific of total or active microbial communities, the DNA- and cDNA-based fingerprints showed that 78% of the amplicons detected were common to both total and active bacteria. This suggests the presence of a bacterial core made of abundant and active bacteria that are essential for feed fermentation within the rumen. However, the different amplicons were distributed differently between diets, suggesting that total and metabolically active bacterial communities have different structures. By contrast, 10% of amplicons were specific to total bacteria and may represent inactive or dead cells; whereas 12% were only found within the active community and may constitute slow-growing bacteria with high metabolic activity. Similarly, excluding the 377-bp amplicon that was found in both present and active bacterial communities, the other indicator species identified were different, depending on whether the analyses were performed on DNA or cDNA samples. Collectively, these results show that cDNA-based methods are more discriminative and these techniques should be more widely used to better characterize rumen microbial populations actively involved in fermentation processes.

## Supporting Information

Table S1 **Ingredient and chemical (g/kg of dry matter) of the experimental diets.**
(DOCX)

Table S2 **Primers sequence used to target the** ***rrs*** **and** ***mcrA*** **genes.**
(DOC)

Table S3 **Amplification programs used for qPCR and LH-rrs.**
(DOC)
