# Supplementary material for: Diet-Induced Alterations in Total and Metabolically Active Microbes within the Rumen of Dairy Cows
Source: PLoS One. 2013 Apr 10;8(4):e60978. doi: 10.1371/journal.pone.0060978 (PMC3622600; doi:10.1371/journal.pone.0060978)
Supplement: Table S1 — Ingredient and chemical (g/kg of dry matter) of the experimental diets. (DOCX) [file pone.0060978.s001.docx]

**Table S1. Ingredient and chemical (g/kg of dry matter) of the experimental diets**

|  | Treatment | | |
| --- | --- | --- | --- |
|  | 0% CS | 50% CS | 100% CS |
| *Ingredient* |  |  |  |
| Barley silage | 544 | 272 | - |
| Corn silage | - | 272 | 544 |
| Barley grain, rolled | 213 | 107 | - |
| Corn grain, ground | - | 52 | 106 |
| Corn gluten feed | - | 57 | 113 |
| Soybean meal | 148 | 130 | 111 |
| Timothy Hay | 55 | 55 | 55 |
| Soybean hulls | - | 23 | 46 |
| Rumen inert fat | 19 | 9 | - |
| Urea | - | 2 | 4 |
| Mineral and vitamin supplement | 21 | 21 | 21 |
| *Chemical composition* |  |  |  |
| Organic matter | 929 | 936 | 945 |
| Crude protein | 167 | 161 | 159 |
| Acid detergent fiber | 244 | 221 | 186 |
| Starch | 166 | 206 | 256 |
| Crude fat | 63 | 58 | 56 |
