# Supplementary material for: Diet-Induced Alterations in Total and Metabolically Active Microbes within the Rumen of Dairy Cows
Source: PLoS One. 2013 Apr 10;8(4):e60978. doi: 10.1371/journal.pone.0060978 (PMC3622600; doi:10.1371/journal.pone.0060978)
Supplement: Table S2 — Primers sequence used to target the rrs and mcrA genes. (DOC) [file pone.0060978.s002.doc]

**Table S2. Primers sequence used to target the *rrs* and *mcrA* genes**

| Target | Primer set | Primer sequence 5' - 3' | Used for | Source |
| --- | --- | --- | --- | --- |
| *Bacteria* | 520-F | AGC AGC CGC GGT AAT | qPCR |  |
|  | 799- R2 | CAG GGT ATC TAA TCC TGT T | qPCR |  |
| *Prevotella* spp. | gPrevo-F | CAC RGT AAA CGA TGG ATG CC | qPCR |  |
|  | gPrevo-R | GGT CGG GTT GCA GAC C | qPCR |  |
| *Fibrobacter succinogenes* | FibSuc3-F | GCG GGT AGC AAA CAG GAT TAG A | qPCR |  |
|  | FibSuc3-R | CCC CCG GAC ACC CAG TAT | qPCR |  |
| *Archaea*  (*rrs*) | 1174-F | GAG GAA GGA GTG GAC GAC GGT A | qPCR |  |
|  | 1406-1389-R | ACG GGC GGT GTG TGC AAG | qPCR |  |
| *Archaea*  (*mcrA*) | qmcrA-F | TTC GGT GGA TCD CAR AGR GC | qPCR |  |
|  | qmcrA-R | GBA RGT CGW AWC CGT AGA ATC C | qPCR |  |
| Protozoa | 316-F | GCT TTC GWT GGT AGT GTA TT | qPCR |  |
|  | 539-R | CTT GCC CTC YAA TCG TWC T | qPCR |  |
| *Bacteria* | 27-F | **6FAM**-AGA GTT TGA TCM TGG CTC AG | LH-PCR |  |
|  | 355-R | GCT GCC TCC CGT AGG AGT | LH-PCR |  |
