# Supplementary material for: Diet-Induced Alterations in Total and Metabolically Active Microbes within the Rumen of Dairy Cows
Source: PLoS One. 2013 Apr 10;8(4):e60978. doi: 10.1371/journal.pone.0060978 (PMC3622600; doi:10.1371/journal.pone.0060978)
Supplement: Table S3 — Amplification programs used for qPCR and LH-rrs. (DOC) [file pone.0060978.s003.doc]

**Table S3. Amplification programs used for qPCR and LH-*rrs***

| Assay | Target | Initial heating | Denaturation | Annealing/Elongation | Final  extension | E^1^  cDNA / DNA |
| --- | --- | --- | --- | --- | --- | --- |
| qPCR | *Bacteria* | 95°C, 10 s | 95°C, 15 s | 58°C, 1 min | - | 98 / 96 |
|  | *Prevotella* spp*.* | 95°C, 10 s | 95°C, 15 s | 55°C, 5 s then 72°C, 30s | - | 91 / 90 |
|  | *F. succinogenes* | 95°C, 10 s | 95°C, 15 s | 60°C, 30 s | - | 97 / 98 |
|  | *Archaea* (*rrs*) | 95°C, 10 s | 95°C, 15 s | 63°C, 15 s then 72°C, 30 s | - | 97 / 98 |
|  | *Archaea* (*mcrA*) | 50°C, 2 min then 95°C, 1 min | 95°C, 15 s | 60°C, 1 min |  | 91 / 92 |
|  | Protozoa | 95°C, 10 s | 95°C, 15 s | 60°C, 30 s | - | 96 / 95 |
| LH-*rrs* | *Bacteria* | 94°C, 2 min | 94°C, 1 min | 55°C, 1 min then 72°C, 1 min | 72°C, 20 min | - |

^1^ PCR efficiencies in % obtained for cDNA and DNA samples
